# An Investigation for Future Practice of Elective Hip and Knee Arthroplasties during COVID-19 in Romania

**DOI:** 10.3390/medicina59020314

**Published:** 2023-02-08

**Authors:** Flaviu Moldovan, Adrian Gligor, Liviu Moldovan, Tiberiu Bataga

**Affiliations:** 1 Department of Orthopedics-Traumatology, “George Emil Palade” University of Medicine, Pharmacy, Science, and Technology of Targu Mures, 38 Gh. Marinescu Street, 540142 Targu Mures, Romania; 2 Biomedical Research Center, “George Emil Palade” University of Medicine, Pharmacy, Science, and Technology of Targu Mures, 1 Nicolae Iorga Street, 540088 Targu Mures, Romania; 3 Faculty of Engineering and Information Technology, “George Emil Palade” University of Medicine, Pharmacy, Science, and Technology of Targu Mures, 1 Nicolae Iorga Street, 540088 Targu Mures, Romania

**Keywords:** orthopedics, COVID-19, coronavirus, pandemic, elective hip surgery, elective knee surgery, surgical volume, arthroplasty trends, length of hospital stay

## Abstract

*Background and Objectives*: Elective arthroplasty in Romania has been severely affected by the COVID-19 pandemic, and its effects are not quantified so far. The aim of this paper is to determine the impact of COVID-19 on arthroplasty interventions and how they varied in Romania. *Materials and Methods*: We performed a national retrospective analysis of patients who underwent primary and revision elective hip and knee interventions at the 120 orthopedic-traumatology hospitals in Romania that are registered in the National Endoprosthesis Registry from 1 January 2019 to 1 September 2022. First, we examined the monthly trend in the number of surgeries for seven categories of arthroplasties. We calculated the percentage change in the average number of cases per month and compared them with other types of interventions. We then examined the percentage change in the average monthly number of arthroplasty cases, relative to the number of COVID-19 cases reported nationwide, the influence of the pandemic on length of hospital stay, and the percentage of patients discharged at home who no longer follow recovery protocols. Finally, we calculated the impact of the pandemic on hospital revenues. *Results*: There was an abrupt decrease in the volume of primary interventions in hip and knee patients by up to 69.14% with a low degree of patient care, while the average duration of scheduled hospitalizations increased. We found a 1–2-day decrease in length of hospital stays for explored arthroplasties. We saw an increasing trend of home discharge, which was higher for primary interventions compared to revision interventions. The total hospital revenues were 50.96% lower in 2020 compared to 2019, and are currently increasing, with the 2022 estimate being 81.46%. *Conclusions*: The conclusion of this study is that the COVID-19 pandemic severely affected the volume of arthroplasty of the 120 hospitals in Romania, which also had unfavorable financial implications. We proposed the development of new procedures and alternative clinical solutions, as well as personalized home recovery programs, to be activated if necessary, for possible future outbreaks.

## 1. Introduction

The COVID-19 pandemic began in late 2019 in Wuhan China, and it spread to most countries in the world, including Romania. Due to the massive increase in the number of people infected with coronavirus in Romania, on 16 March 2020, a state of emergency was decreed in Romania. By the end of December 2020, there were over half a million confirmed COVID-19 infections in Romania [1], and by mid-October 2021, there were almost 20,000 infections and over 500 deaths daily [2]. The alert ceased on 9 March 2022.

The rapid implementation of control measures largely prevented an increase in COVID-19 cases, and this alleviated the overload of the health system, which had been highly strained since the early stages of the pandemic [3].

Due to the COVID-19 pandemic, most international arthroplasty practices have undergone significant changes. An online survey for orthopedic surgeons was administered during the COVID-19 pandemic, and its results indicate a near-complete reduction in joint arthroplasties [4]. Another survey conducted in 32 countries shows that orthopedic surgery has been severely affected by the pandemic; more than one third of surgeons have closed their offices, and 70% of them have not scheduled elective interventions [5].

Severe reduction in the number of surgeries due to the pandemic has had unpleasant consequences for patients, causing significant psychosocial distress [6] and deterioration in the health of patients whose elective procedures were delayed [7]. One quarter of patients reported experiencing significant physical and emotional impairments [8]. Patients suffering from hip and knee arthritis pain have continued to struggle with end-stage pain and suffer from anxiety related to the coronavirus disease. Ninety percent of them want to have surgery as soon as possible [9]. The severe reduction in arthroplasty volume due to the COVID-19 pandemic has had a profound impact on patients and generated substantial financial losses for hospitals [10].

The pandemic also had a severe disruptive effect on orthopedic surgery in Romania. A major turning point was in March 2020, when orthopedic activity stopped entirely, and it was reduced to urgent cases only in most clinics in Romania [11]. Cancellation of elective surgery to control the COVID-19 pandemic had significant effects on the volumes of hip and knee arthroplasty in Romania. Surgeons had to manage patients without knowing their infection status, postponing elective surgeries. The decrease of activity in hospitals was also caused by Intensive Care Units being occupied by patients with severe biological statuses due to COVID-19, who needed ventilation and were intubated. Due to the epidemiological situation, many resources of this medical facility were closed.

All elective surgery procedures, primary arthroplasty, revision of hip and knee surgeries, and bone transplants were postponed, resulting in an unfair situation for these patients and creating many difficulties in their economic and social lives.

The recommendation to postpone surgery came from the Romanian Ministry of Health, but the final decision in all cases was made by surgeons and hospital administrations, who considered the postponements necessary to save hospital resources and focus on treating patients with COVID-19. These decisions have resulted in a significant decrease in the number of cases of elective orthopedic surgery, which required additional treatment and increased social and economic costs. Hip and knee joint replacement procedures have seen an increase in the number of interventions since October 2020.

There is currently a tendency to return to normalcy, which is slow and may be different from what we were used to, but a transition to a more sustainable healthcare reality should be planned to accommodate a COVID-19 world [12]. We need to prepare for difficult ethical decisions regarding the resumption of elective surgery. Future decisions in this matter should be supported by reliable data, expert recommendations and regional circumstances [13].

Based on the research trends identified in the bibliographic literature and the data collected for the studied interventions, we formulated the following study hypotheses:

**Hypothesis 1 (H1).**  *During the pandemic, elective procedures of hip and knee decreased, and their return in the current period of 2022 is below the level before the pandemic, 2019–16 March 2020.*

**Hypothesis 2 (H2).**  *During the pandemic, primary elective hip and knee procedures recorded a larger percentage decrease in volume in comparison with other types of surgical procedures performed in hospitals for emergency and nonemergency situations.*

**Hypothesis 3 (H3).**  *There has been a positive relationship between COVID-19 per capita rates in Romania and a monthly decrease in arthroplasties since March 2020.*

**Hypothesis 4 (H4).**  *The average length of a hospital stay decreased by case type after March 16,2020, to allow hospitals to provide enough capacity for COVID-19 cases, and currently the average length of hospital stays is increasing.*

**Hypothesis 5 (H5).**  *Even though the number of surgeries performed in relation to the number of recommended outpatient surgeries decreased, the scheduled duration of interventions increased during the pandemic.*

**Hypothesis 6 (H6).**  *COVID-19 led to an increase in at-home discharged rates compared to a post-acute care provider due to fears of increased transmission rates in an institutional setting compared to a home setting.*

**Hypothesis 7 (H7).**  *There is a direct correlation between the number of interventions and the hospital’s income.*

Given the severe impact on orthopedic surgery, this study aims at a good understanding of the effects of the COVID-19 pandemic on elective orthopedic surgery in Romania. Another aim, which has not been evaluated so far, is to determine the impact at the beginning of the pandemic and how this response varied during the pandemic, as well as the current trends of return to normalcy. It also aims to evaluate the effects on the length of hospitalization for the surgeries performed during this period, as well as the condition of the inpatient versus outpatient. Finally, we sought to determine the financial implications of this period.

## 2. Materials and Methods

The methodology of this study consisted of:

1. Study design and selection of study participants;
2. Selection of the variables explored in the study and calculus methodology;
3. Data collection and statistical analysis.

### 2.1. Study Design and Participants

The methodology of this study consisted of a retrospective analysis of the patients’ beneficiaries of primary and revision elective hip and knee surgery from 120 orthopedic-traumatology hospitals/departments in Romania. All patients, regardless of gender, who benefited from elective hip and knee interventions, namely: cemented hip, uncemented hip, partial hip, hybrid and reverse hybrid, cemented knee, hip revision, and knee revision, were recruited and included in the study.

For this we used 100% the data on payment requests for health care services for arthroplasty operations between 1 January 2019 and 1 September 2022 with the support of the data recorded in the National Endoprosthesis Registry [14]. A number of 53,748 subjects were included in the studies, who were diagnosed in the outpatient ward with the recommendation of elective surgery and who followed this intervention. Table 1 shows the gender of the patients participating in the study, of which 20,149 (37.49%) were men and 33,599 (62.51%) were women.

The study was approved by the Emergency County Clinical Hospital, from Targu Mures (ECCH).

### 2.2. Evaluation Variables and Methodologies

Based on the formulated hypotheses, we have defined variables and have developed evaluation methodologies appropriate to the following seven explored issues: the volumes of hip and knee elective procedures, comparison with other types of surgical procedures, influence of COVID-19 on the volumes of arthroplasty, length of hospital stay, duration of interventions, hospital discharge rates, hospital revenues.

#### 2.2.1. Elective Hip and Knee Surgery Volume

In order to estimate the volume of elective procedures of hip and knee, we selected as variables the monthly number of the following 7 categories of elective hip and knee procedures: cemented hip (n_ch_), uncemented hip (n_uh_), partial hip(n_ph_), hybrid and reverse hybrid (n_hrh_), cemented knee (n_ck_), hip revision (n_hr_), and knee revision (n_kr_).

#### 2.2.2. Comparison with Other Types of Surgical Procedures

In order to compare the hip and knee operations with other types of surgical procedures performed among hospitalized patients, intervention for nonemergency situations is further analyzed: spinal segmental implants and hemicolectomy, but also procedures performed in emergency situations—i.e., post-fracture osteosynthesis procedures in the femur, coronary artery bypass grafting, coronary angioplasty.

We calculated the annual averages of the 5 types of primary interventions studied: cemented hip (AVn_ch_), uncemented hip (AVn_uh_), partial hip (AVn_ph_), hybrid and reverse hybrid (AVn_hrh_), cemented knee (AVn_ck_), for the years 2019, 2020, 2021 and 2022 until 1 September. The variables explored are the percentage changes in the average monthly cases for each of the primary types of cases analyzed in the years 2020, 2021 and 2022 until 1 September, compared to 2019. For example, for the cemented hip intervention in 2020 compared to 2019, we computed the ratio 100 * (AVn_ch_^2020/^AVn_ch_^2019^-1).

Then we computed the corresponding annual averages for spinal segmental implants (AVn_ss_), hemicolectomy (AVn_he_), post-fracture osteosynthesis procedures in the femur (AVn_pf_), coronary artery bypass grafting (AVn_cb_), coronary angioplasty (AVn_ca_). For these surgeries we also explored the percentage change in the average monthly cases in the same period, compared to 2019.

At the end, the percentage decreases of the two categories of procedures, primary interventions for hip and knee versus other types of common surgeries, were compared.

#### 2.2.3. The Influence of COVID-19 on the Volumes of Arthroplasty Interventions

In this situation, we examined the percentage change in the average monthly number of arthroplasty cases, compared to the number of COVID-19 cases in Romania, published in the official statistics.

We calculated the total volume of surgical operations TS = n_ch_ + n_uh_ + n_ph_ + n_hrh_ + n_ck_, as the sum of the 5 types of primary interventions: cemented hip (n_ch_), uncemented hip (n_uh_), partial hip (n_ph_), hybrid, and reverse hybrid (n_hrh_), cemented knee (n_ck_).

The variables explored are the percentage changes in the monthly total volume of primary interventions, in the interval March 2020–March 2022. For example, in the case of primary intervention in April 2020 compared to March 2020, the ratio computed was: 100 ∗ (TS^April 2020/^TS^March 2020^ − 1).

#### 2.2.4. Length of Hospital Stay

The variables explored in this study are the average hospitalization durations for the following four groups of surgeries: cemented hip (hd_ch_)—uncemented hip (hd_uh_)—cemented knee (hd_ck_); partial hip (hd_ph_); hybrid and reverse hybrid (hd_hrh_); revision hip (hd_hr_)—revision knee (hd_kr_). In the first group we included three types of interventions, and in the fourth group we included two types of interventions, because before the pandemic these hospital stays were close in number. We graphically represented the average hospitalization duration by month of surgery for the four groups of surgeries.

For this study, we examined the impact of COVID-19 on the length of hospitalization of the patient for cases of primary and revision arthroplasties, in the interval 1 January 2019–1 September 2022.

#### 2.2.5. The Number of Interventions in Relation to Scheduling Duration

We evaluated for arthroplasty cases whether COVID-19 influenced the percentage of outpatients versus inpatients and the duration for scheduling intervention.

For this we used the number of patients who presented monthly on an outpatient basis and for whom surgery was recommended, for each of the 7 types of elective interventions: cemented hip (n_ch_^ow^), uncemented hip (n_uh_^ow^), partial hip (n_ph_^ow^), hybrid and reverse hybrid (n_hrh_^ow^), cemented knee (n_ck_^ow^), hip revision (n_hr_^ow^), and knee revision (n_kr_^ow^). Based on the above, we determined the total number of recommended interventions in outpatient wards to be NRS^ow^ = n_ch_^ow^+ n_uh_^ow^ + n_ph_^ow^ + n_ar_^ow^ + n_ck_^ow^ + n_hr_^ow^ + n_kr_^ow^. The number of operations performed each month for the 7 types of elective interventions is known as NPS = n_ch_+ n_uh_ + n_ph_ + n_ar_ + n_ck_ + n_hr_ + n_kr_.

In continuation we defined the first variable of this study according to the ratio between the total number of recommended interventions and the number of operations performed, as Ratio = NPS/NRS^ow^.

The second variable explored is the average scheduling period of the 7 procedures explored, calculated as a monthly average of the time from the date of recommending it in the outpatient ward until it was performed, for the cases recorded in our study.

#### 2.2.6. Outpatients at Home

Next, we looked at whether COVID-19 affected the percentage of primary and revision arthroplasty cases that were discharged at home. In this regard, we used the number of cases discharged at home for each of the 7 types of elective interventions: cemented hip (n_ch_^dh^), uncemented hip (n_uh_^dh^), partial hip (n_ph_^dh^), hybrid and reverse hybrid (n_hrh_^dh^), cemented knee (n_ck_^dh^), hip revision (n_hr_^dh^), and knee revision (n_kr_^dh^). Then we calculated the number of primary cases discharged at home n_p_^dh^ = n_ch_^dh^ + n_uh_^dh^ + n_ph_^dh^ + n_ar_^dh^ + n_ck_^dh^, respectively, the number of revision cases discharged at home nr^dh^ = n_hr_^dh^ + n_kr_^dh^. The variables of this study are the average monthly percentage of cases discharged at home for primary interventions, which is the ratio 100 ∗ np^dh^/(n_ch_ + n_uh_ + n_ph_ + n_ar_ + n_ck_), respectively, for revision interventions, which is the ratio 100 ∗ nr^dh^/(n_hr_ + n_kr_).

#### 2.2.7. Hospital Revenues

Finally, we calculated the impact of the revenue on the hospital. For this, we calculated the total costs of the primary and revision interventions performed, in each of the years 2019, 2020, 2021, respectively, 2022 until 1 September, as a product of the number of surgical interventions performed and the average cost per unit. The two variables of the study are the absolute values of the incomes realized in these years, as well as the comparison with the incomes of 2019 of the annual incomes realized from the medical care of the arthroplasty cases after 2019, until 1 September 2022.

### 2.3. Data Collection and Statistical Analysis

In the period June–October 2022, data were collected from the National Endoprosthesis Registry on the total number of beneficiaries of elective primary and revision surgeries from the 120 orthopedic-traumatology hospitals/departments in Romania, after which their statistical processing was performed. The data were collected, preprocessed and filtered in Microsoft Excel, then transferred to GNU PSPP and Matlab for further processing.

For the elective hip and knee procedures: cemented hip, uncemented hip, partial hip, hybrid and reverse hybrid, cemented knee, hip revision, and knee revision, we examined the monthly trend in the number of surgeries in 2019 (Table 2), 2020 (Table 3), 2021 (Table 4), and 2022 until 1 September (Table 5).

We also collected the number of patients who presented monthly on an outpatient basis and who had surgery recommendations, for each of the 7 types of elective interventions, from which by combining we obtained the total number of recommended interventions in outpatient wards at ECCH. Then, for the patients who underwent one of the 7 surgical interventions studied, we collected the duration of hospitalization of the operated patient and the number of cases discharged at home.

At the end, from the financial reports of the hospitals, we extracted the average costs of the 7 types of elective interventions that we used in the economic calculation regarding the revenues recorded by the hospitals.

The methodology for processing the collected data consisted of a separate analysis for each of the seven studied issues.

In the analysis of the volumes of elective procedures of hip and knee, as there was a strong effect of the month of the year, we also calculated the three-month moving average (MA) of the number of cases of each of the 7 types of elective procedures of hip and knee to more easily examine any potential time trend during the quarter. In the analysis of the length of the operated patient’s hospital stay we calculated the average values of the hospitalization durations.

Statistical analysis was performed with Statistics and Machine Learning Toolbox Version 12.3 from Matlab R2022a (The Math Works, Inc., Natick, MA, USA).

## 3. Results

In our study we relied on reports from the 125 orthopedic-traumatology hospitals/departments in Romania, which, according to the current regulations of the Ministry of Health report on a regular basis their endoprosthetics activity (hip, knee and spinal segmental implants) to the National Endoprosthesis Register [14]. It was excluded from the list of five hospitals (4%), which do not perform hip and knee interventions, only spinal segmental implants, or that did not provide complete information. In the end, information received from the 120 hospitals was included in the study, which represents a proportion of 96% of the orthopedic-traumatology hospitals/departments existing at the national level. This is a representative sample for the study group consisting of patients at the hospitals included in the study (95% CL, CI = 1221.55 ± 45.38, ±5% acceptable margin of error),which ensures that the study is not underpowered.

**Figure 1:**
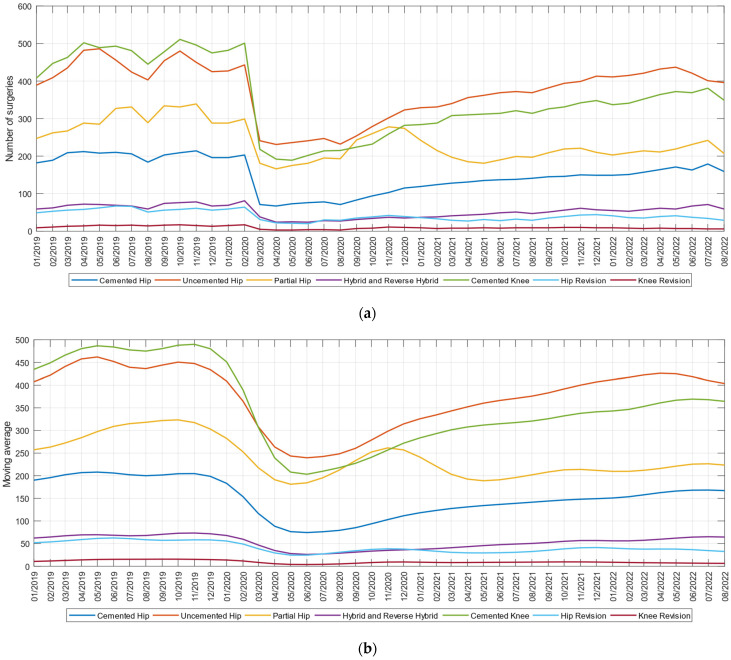
(**a**) Monthly volume; and (**b**) 3-month moving average in period 1 January 2019–1 September 2022: cemented hip, uncemented hip, partial hip, hybrid and reverse hybrid, cemented knee, revision knee, and revision hip.

In the study we examined the monthly trend in the volume of cases between 1 January 2019 and 1 September 2022, for primary procedures: cemented hip, uncemented hip, partial hip, hybrid and reverse hybrid, cemented knee, respectively, for revision procedures: revision hip and revision knee. As shown in Figure 1a, the number of primary and revision surgeries declined sharply since March 2020. The variables studied indicate average monthly decreases of up to 55.47% for primary interventions and 69.14% for revision interventions in April 2020, compared to the volumes of interventions performed before the pandemic, in February 2020. During 2021, the number of operations performed increased slightly, being at a monthly volume of 71.80% compared to the pre-pandemic period. There is also an increase in 2022 until 1 September, the number of monthly interventions being approximately 80.79% of those prior to the pandemic. These trends are even clearer in the three-month moving average view in Figure 1b, in which the effect of the month of the year was smoothed out.

A two-sided Wilcoxon rank sum test applied for data related to consecutive years of elective procedures with computed values of *p_19–20_* = 0.0004, *p_20–21_* = 0.0141, *p_21–22_* = 0.00014 and *p_19–22_* = 0.00015 indicates the rejection of the null hypothesis of equal medians at the default 5% significance level. This confirms the H1 hypothesis that during the pandemic the elective procedures of hip and knee have decreased, and the return in the current period of 2022 is below the level before the pandemic.

In order to examine the percentage change in the volume of cases for the primary procedures, we compared the average monthly volume in 2019, 2020, 2021 and 2022 until 1 September (Table 6).

The study variables indicate that the primary procedures that recorded the largest decreases in 2020 compared to 2019 are cemented hip (−49.2%), hybrid and reverse hybrid (−45.1%), cemented knee (−43.6%), compared to the other two primary interventions uncemented hip and partial hip, in which the decreases were below −35%.

If we compare the primary orthopedic interventions with other types of procedures performed in the hospitals, we find that they were less affected, the largest decrease being for hemicolectomy interventions (−32.9%), in nonemergency situations and coronary artery bypass grafting (−34.4%) in emergency situations. During 2021, the decrease in the volume of cases compared to 2019 is smaller, the most severely affected being the cemented knee surgeries (−33.2%), while for other types of surgeries the most affected were spinal segmental implants (−37.7%). The 2022 prevision for the variation in the volume of cases indicates that partial hip (−27.4%) is the most affected, while other types of spinal segmental implants are located with a gap of (−37.5%) compared to the pre-pandemic period. This study partially confirms the H2 hypothesis that during the pandemic, primary elective hip and knee procedures recorded a larger percentage decrease in volumes compared to other types of surgical procedures performed in hospitals, but it is found that in the current period the nonemergency spinal segmental implant procedures are more affected.

**Figure 2:**
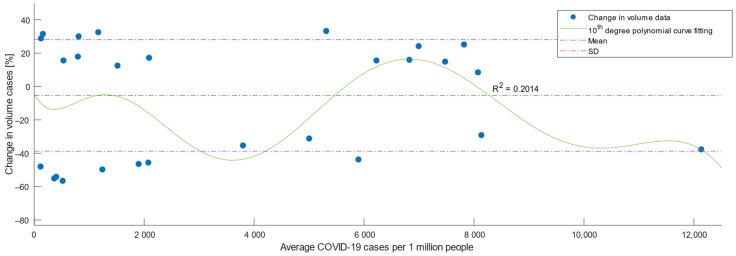
The scatterplot for variation of elective intervention volumes and the number of COVID-19 cases during the pandemic period in Romania. A polynomial curve fitting with an R square has been added to the scatterplot in order to show the correlation.

Third, we examined the variation of the percentage decrease in the volume of primary and revision arthroplasty in relation to the number of cases of COVID-19. Figure 2 is a scatterplot showing on the Y axis the percentage variation of arthroplasty cases in the analyzed periods and on the X axis, the number of COVID-19 cases in the country during the analyzed period March 2020–March 2022. As indicated by the polynomial curve fitting by using ordinary least squares estimation and the calculated value R^2^ = 0.2014, a higher number of COVID-19 case rates was correlated with a higher percentage decrease in elective arthroplasties, as values for R^2^ below the threshold of 0.5 indicate a low level of correlation [15]. However, the rate of COVID-19 cases was loosely associated with the decrease, explaining only 20% of the variation at the study population level, as well as the study hypothesis H3.

Next, we examined the impact of COVID-19 on the length of hospital stay, on four groups of surgeries consisting of cemented hip-uncemented hip-cemented knee, partial hip, hybrid and reverse hybrid, and revision knee-revision hip.

**Figure 3:**
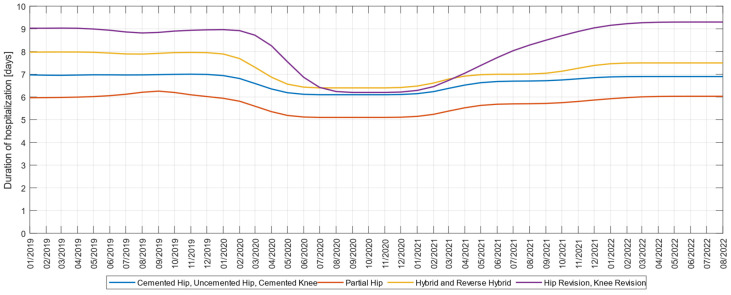
Three-month moving average of the monthly average length of hospital stays for: cemented hip-uncemented hip-cemented knee, partial hip, hybrid and reverse hybrid, revision knee-revision hip, for the interval 1 January 2019–1 September 2022.

We plotted a three-month moving average time trend of these metrics as seen in Figure 3. For cemented hip-uncemented hip-cemented knee interventions, we noticed for the variables explored, a slight decrease in the length of hospital stay from seven days before the pandemic to 6.1 days during the onset of the pandemic, and in 2022 it increased to 6.9 days. A similar decrease occurred in hospitalization for hybrid and reverse hybrid interventions, from eight days before the pandemic to 6.4 days during the pandemic, and in 2022 reaching 7.5 days. Also, for partial hip interventions, the duration of hospitalization decreased from 6.2 days to 5.2 days during the pandemic. The most significant decrease was recorded in knee-revision and hip-revision prosthesis interventions, from 9.2 days before the pandemic to 6.1 days during the pandemic, so that in 2022 the length of hospitalization would return to pre-pandemic values.

Corresponding to the type of primary interventions, the hospitalization period before the pandemic was 6–9 days, which during the pandemic decreased to 5–7 days; that value now tends to return to pre-pandemic values, as assumed in our H4 hypothesis.

**Figure 4:**
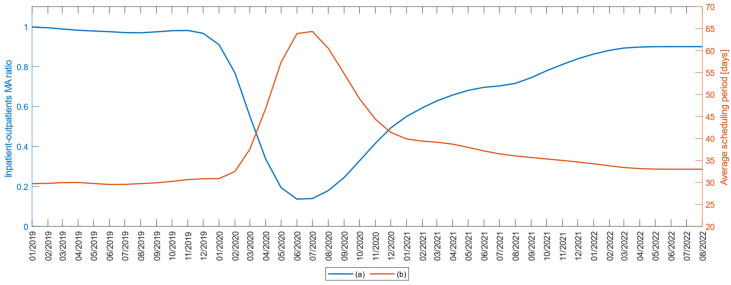
(**a**) The three-month moving average of the ratio of inpatients to outpatient patients; (**b**) The three-month moving average of the average length of hospital programming.

To examine the degree of patient care among those who received primary and review interventions, each month of the explored interval 1 January 2019–1 September 2022 was represented by the ratio between the total number of recommended interventions and the number of operations performed (Figure 4a). In the years 2019 and the first quarter of 2020, this ratio has the value 1; during the pandemic period the ratio decreased to 0.1, so that in 2022 it reaches 0.9.

At the same time, the second variable of the study, the average duration of hospitalization scheduling, which was 30 days before the pandemic, was extended to 64 days in the pandemic onset period, so that in 2022, it reached 34 days (Figure 4b).

The represented variations of the two variables in this study confirm the H5 hypothesis regarding the increase of the interventions’ scheduled duration during the pandemic, even if the numbers of surgeries have decreased.

The traditional model of care for recovery from hip and knee replacement involves intensive in-person outpatient physical therapy followed by recovery at home. Due to the pandemic, the need for social distancing arose, which meant avoiding direct contact between health personnel and patients. But closed sanitary spaces are often crowded. With the significant increase in the number of cases of COVID-19 and the reduction in the number of elective interventions, the clinical physiotherapeutic was severely limited, in terms of the number of patients, and the duration of recovery sessions. Consequently, the need to outsource recovery programs through virtual physical therapy and telerehabilitation was outlined.

**Figure 5:**
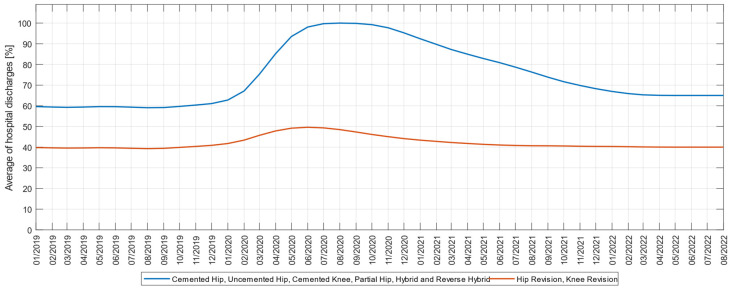
Three-month moving average of the average monthly percentage of cases discharged at home for cemented hip-uncemented hip-cemented knee-partial hip-hybrid and reverse hybrid, respectively revision knee-revision hip, in the interval 1 January 2019–31 March 2022.

In researching the effects of the pandemic on discharge rates, we plotted the cumulative percentage discharged directly at home compared to a post-acute care provider, for the two types of interventions studied, primary and revision arthroplasty, respectively. Analysis of these variables indicates that all primary interventions have been on an upward trend since the second half of March 2020 as seen in Figure 5. If before the pandemic the percentage of discharges directly at home was 60%, during the pandemic it reached 100%, after which it fell again in 2022 to 65%, which, regarding primary arthroplasties confirms the H6 hypothesis during the pandemic and establishes the tendency to return.

Interestingly, for pre-pandemic revision interventions, the percentage of direct discharge at home was 40%; during the pandemic it increased to 50%, and in 2022 it returned to 40%. This partial confirmation of hypothesis H6 in the case of revision arthroplasties can be explained by the degree of awareness of patients undergoing revision interventions and who know the importance of recovery processes through physical therapy, compared to those who undergo primary interventions.

**Figure 6:**
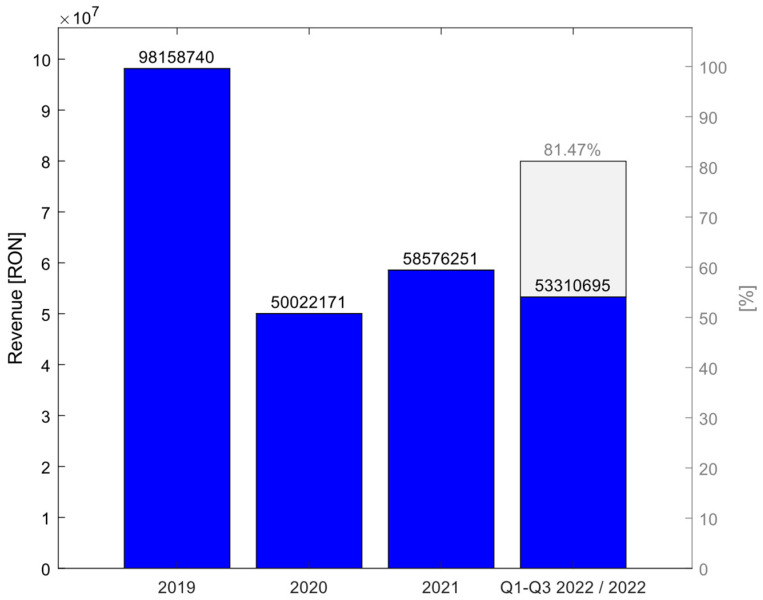
Hospitals revenues between 1 January 2019 and 1 September 2022.

Finally, we calculated the impact of COVID-19 on hospital revenues. The exploration of the study variables shows that for primary and revision interventions, compared to 2019, in 2020 revenues stood at 50.96%; in 2021 they increased to 59.96%, and in the first eight months of 2022 they represent 44.73% of 2019, which by extrapolation for similar values for the rest of the year would represent 81.46% of 2019 revenues (Figure 6). These findings confirm the H7hypothesis of the study regarding the direct correlation between hospital income and the volume of surgeries performed.

## 4. Discussion

The pandemic has affected the lifestyle of the population, impacting normal daily activities, but also regular surgical treatments in patients with osteoarthritis of the hip and knee. The COVID-19 pandemic had a significant impact on clinical arthroplasty interventions, with personal and financial consequences for patients and physicians, and a drastic reduction in elective hip and knee surgery.

The results of our study suggest that elective hip and knee surgery during the COVID-19 pandemic decreased significantly, and the impact of the pandemic on the volume of surgery cases in Romania was rapid and dramatic, as the Ministry of Health recommended stopping elective procedures. Our study found a decrease of up to 55.47% for primary interventions and 69.14% for revision interventions without being completely reduced, which can be explained by the fact that due to the increased risk of morbidity these interventions are considered urgent, such as recurrent hip dislocation or prosthetic joint infection.

These findings are consistent with research by Kazubski et al. [16] showing that the COVID-19 pandemic resulted in fewer hip and knee replacement procedures. According to a study by Simon et al. [17], the total number of primary and revision joint arthroplasties in 2020 compared to 2019 decreased by 86%. Global studies show that the COVID-19 pandemic has caused significant delays in hip and knee surgical procedures reported in countries such as Germany [18], Scotland [19], Poland [20,21], the USA [15] and Taiwan [22], where a 20%–30% reduction in operative volume is attributed to patient fears. This is also consistent with the findings of our study showing that the percentage decrease in arthroplasty volume can be explained to a limited extent by the number of COVID-19 cases. In these conditions, surgeons and health managers should consider delays in surgical treatment of cases [23], by assessing the risk versus the potential harm that may occur from delaying intervention [24], given the lack of fear of infection risk on the part of surgeons [25], but also given the good behavior of patients who have had surgery performing the Polymerase Chain Reaction (PCR) tests [26].

In our study, we found that with the “flattening of the pandemic curve” in the course of 2022, elective operations have gradually resumed, and primary elective hip and knee procedures are experiencing greater increases than other nonemergency procedures such as spinal segmental implants. This brought to the center of the debate the assurance of the operations’ safety [27] and the demanding monitoring of the interventions [28]. The process appears to be more difficult for patients than for surgeons, as there is some reluctance in patients to reschedule canceled surgeries [29]. Preoperative assessment of patients based on a standardized questionnaire and an algorithm regarding COVID-19 can increase the accuracy of the decision to perform the operation [30]. Another approach to patient selection, rationalization of resources, and financial implications is to examine them in relation to the five tenets of bioethics: beneficence, maleficence, autonomy, and justice, as shown in the study by Moses et al. [31].

In our study, we observed the increase in the scheduled duration of interventions during the pandemic from 30 to 64 days, even though the number of surgical interventions performed in relation to intervention recommendations decreased by 90%. However, we found a decrease in the length of hospital stays in cases of elective procedures due to the pandemic, which, according to the type of intervention, reached 5–7 days during the pandemic, compared to the pre-pandemic period in which the same interventions required hospital stays of 6–9 days. In a cohort study, Roger et al. [32] show that the length of hospitalization after primary hip or knee arthroplasty depends on diabetes, the day of surgery, and the destination of discharge, and our study may add the inverse proportional dependence on the COVID-19 pandemic. At a medical center in Poland, the COVID-19 pandemic also had a marked effect on the average length of hospital stay and the ratio of male to female patients [33]. We also found a greater degree of awareness of patients undergoing revision interventions compared to those undergoing primary interventions regarding the importance of recovery processes through physical therapy.

The large number of elective interventions canceled during the pandemic generated major financial losses for health institutions [10,17,34], and our study also highlighted decreases in hospital revenues in Romania of 50.96% in 2020 compared to 2019. Managers and surgeons are constantly required to come up with innovations to provide good quality medical care while reducing costs, a requirement that has been further amplified by the pandemic. One such solution to streamline orthopedic surgical care and eliminate postoperative hospitalization is proposed by Chambers et al. [35] for ambulatory total knee arthroplasty. The impact of postponing elective surgery cases requires arrears recovery planning [36], and with more effective planning, medical systems will no longer be subject to the effects of other pandemics [37]. This required reassessing how hospitals manage their processes and adapting to new business environments for which medicine and education do not provide immunity [38].

Sarac et al. [39] showed that during the COVID-19 pandemic, there were few states that published guidelines specific to orthopedic surgery. After this pandemic, with the support of information about orthopedic practices collected from surgeons around the world, specific guidelines can be developed and adapted to the specific clinical and financial situation of each hospital [40]. Intraoperative and postoperative guidelines [41,42] can better prepare elective surgical specialties for possible further pandemic waves and establish a new paradigm for health care in orthopedics [43]. Concomitantly, as information about the state of the pandemic evolves, surgical managers should update decision-making procedures for performing elective surgery [44,45]. Effective dialogue with religious groups can also improve the outcomes of public health interventions [46].

In this context, telemedicine can replace some of the patient visits in outpatient departments, which would contribute to a substantial reduction in the volume of physical work in hospitals. Telemedicine through virtual consultations has the potential to be integrated into emergency orthopedics in the future [47], but also in clinical education and teaching, for residents and young specialists [48].

The research model developed in this study can be used in a global analysis at an international level. It can also be used as an example of good practice for situations that are similar to those described in the paper. The national situation presented allows further research through meta-studies to make comparisons with similar situations at the international level and to formulate global conclusions.

There are some limitations to this study. A first limitation is the use of the database of the National Endoprosthesis Registry from which we extracted arthroplasty trends in Romania. It is possible that some orthopedic-traumatology hospitals/departments do not accurately or in a timely manner report all arthroplasty procedures performed, which may affect the accuracy of some data presented. However, the database used is useful in determining the trends of performing elective procedures in current practice. Another limitation stems from the existence during this period of change in clinical practice by switching to outpatient care, which was achieved mainly for reasons of economic efficiency, but which due to confusion can be attributed to the COVID-19 pandemic.

Despite these limitations, the study is clinically relevant because it presents the statistical and financial results of the elective operation of the hip and knee, in Romania, during the COVID-19 pandemic. Once all restrictions are lifted, future studies will be able to assess the full effect of the pandemic on elective surgery.

## 5. Conclusions

In conclusion, our analysis of the database of the National Endoprosthesis Registry found that after the onset of the COVID-19 pandemic emergency on 16 March 2020, there were dramatic decreases in elective hip and knee surgery volumes by up to 69.14%, a low degree of patient care, increased average durations for hospitalization scheduling, and a growing trend toward more outpatient cases. Also, after this date, we identified shorter hospital stays, with 1–2 days, a large part of the patients being discharged at home. Compared to 2020, towards the end of 2021 the volume of elective interventions increased by 15.58% a trend that continues this year as well. Due to the decrease in the volumes of elective interventions during this pandemic period, there have been significant clinical and financial effects, which are still not overcome at present, and for which we anticipate clinical and financial effects in the next period as well. Consequently, in order to successfully overcome similar pandemic periods, for the volume and categories of interventions affected, new procedures and alternative clinical solutions should be developed to be activated in case of necessity for the safe performance of arthroplasties.

Orthopedic practice must be modified by adapting to constantly evolving medical realities. For possible future outbreaks it is necessary to develop new procedures and alternative clinical solutions, and in order to achieve a smooth adaptation to volume changes and the disparate needs of surgeons and advancing medical practices, early and open communication is needed. For this, it is necessary to continually collect clinical data, promote a fair and transparent system for prioritizing cases, optimize perioperative workflows, and effectively manage medical equipment and human resources through the psychological training of medical staff. New emergency procedures must separate positive from negative COVID-19 patients, find alternative healthcare facilities to manage a surge of critically ill patients, or isolate asymptomatic but infectious ones. Public-private partnerships should be ratified to expand pandemic preparedness capabilities. It is necessary to build new multifunctional spaces, or to modernize existing ones, so that they can be easily transformed into screening centers or isolation spaces. Orthopedics has to be digitized through the development of telemedicine platforms in which doctors and patients interact through virtual and augmented reality, mediate the communication with the support of artificial intelligence technologies, and preoperative planning is carried out with the support of surgical programming applications and 3D printing technologies.

We also propose the development of personalized home recovery programs, through virtual physical therapy and telerehabilitation in which physical therapy providers perform intensive physical therapy at the patients’ homes. They offer a more compliant recovery path at a lower cost through personalized recovery in the comfort of a patient’s home that is just as effective as the traditional face-to-face recovery.

These conclusions are important because, for the volume of affected arthroplasties that are presented in this study, we can prepare in advance for possible future outbreaks, in which we will know how to behave.

## Figures and Tables

**Table 1:** Gender of patients who participated in the study.

Year	2019		2020		2021		2022	
Gender	Men	Women	Men	Women	Men	Women	Men	Women
Cemented hip	1051	1371	512	718	701	943	582	711
Uncemented hip	2298	2995	1438	2018	1883	2533	1502	1832
Partial hip	1557	2031	1138	1595	1051	1414	782	954
Hybrid and reverse hybrid	358	465	189	264	245	331	217	265
Cemented knee	1313	4375	773	2437	945	2853	716	2149
Hip revision	261	432	176	254	167	239	122	170
Knee revision	64	105	38	52	46	59	24	34

**Table 2:** Number of cases in 2019.

Month of the Year	01	02	03	04	05	06	07	08	09	10	11	12	Total
Cemented total hip (n_ch_)	182	189	209	212	208	210	206	184	203	209	214	196	2422
Uncemented total hip (n_uh_)	389	409	435	482	486	456	424	403	454	480	450	425	5293
Partial hip (n_ph_)	247	262	267	288	285	327	331	289	334	331	339	288	3588
Hybrid and reverse hybrid (n_hrh_)	59	62	69	72	71	69	67	59	74	76	78	67	823
Cemented knee (n_ck_)	408	447	463	502	489	493	481	445	478	511	496	475	5688
Hip revision (n_hr_)	49	53	56	58	62	67	66	51	56	58	61	56	693
Knee revision (n_kr_)	9	11	13	14	16	15	16	14	16	17	15	13	169

**Table 3:** Number of cases in 2020.

Month of the Year	01	02	03	04	05	06	07	08	09	10	11	12	Total
Cemented hip (n_ch_)	196	203	71	67	73	76	78	71	83	94	103	115	1230
Uncemented hip (n_uh_)	427	443	241	231	236	241	247	232	254	279	302	323	3456
Partial hip (n_ph_)	288	299	181	166	175	181	195	193	243	260	278	274	2733
Hybrid and reverse hybrid (n_hrh_)	69	81	38	24	25	24	28	27	31	34	37	35	453
Cemented knee (n_ck_)	482	501	218	192	189	202	214	215	224	232	259	282	3210
Hip revision (n_hr_)	59	64	31	22	21	20	30	29	35	38	42	39	430
Knee revision (n_kr_)	15	17	5	3	3	4	4	3	7	8	11	10	90

**Table 4:** Number of cases in 2021.

Month of the Year	01	02	03	04	05	06	07	08	09	10	11	12	Total
Cemented hip (n_ch_)	119	124	128	131	135	137	138	141	145	146	150	149	1644
Uncemented hip (n_uh_)	329	331	340	356	362	369	372	369	382	394	399	413	4416
Partial hip (n_ph_)	242	215	197	185	181	190	199	197	209	219	221	210	2465
Hybrid and reverse hybrid (n_hrh_)	37	38	41	43	45	49	51	47	51	56	61	57	576
Cemented knee (n_ck_)	284	288	308	310	312	314	321	314	326	331	342	348	3798
Hip revision (n_hr_)	36	33	29	27	31	28	32	29	35	39	43	44	406
Knee revision (n_kr_)	9	7	8	8	9	8	9	9	9	10	10	9	105

**Table 5:** Number of cases in 2022 until 1 September.

Month of the Year	01	02	03	04	05	06	07	08	Total
Cemented hip (n_ch_)	149	151	157	164	171	163	179	159	1293
Uncemented hip (n_uh_)	411	415	421	432	437	421	401	396	3334
Partial hip (n_ph_)	203	209	214	211	219	231	242	207	1736
Hybrid and reverse hybrid (n_hrh_)	55	53	57	61	59	67	71	59	482
Cemented knee (n_ck_)	337	341	352	364	372	369	381	349	2865
Hip revision (n_hr_)	41	36	35	39	41	37	34	29	292
Knee revision (n_kr_)	9	8	7	8	7	7	6	6	58

**Table 6:** Percentage change in average monthly case volume.

		Average Monthly Volume in 2019 [Absolute Values]	Average Monthly Volume in 2020 [Absolute Values]	CHANGE in Case Volume (2)/(1) [%]	Average Monthly Volume in 2021 [Absolute Values]	Change in Case Volume (4)/(1) [%]	Average Monthly Volume in 2022-09.01 [Absolute Values]	Change in Case Volume (6)/(1) [%]
		(1)	(2)	(3)	(4)	(5)	(6)	(7)
Arthroplasty	Cemented hip (AVn_ch_)	201.8	102.5	−49.2%	137.0	−32.1%	161.6	−19.9%
	Uncemented hip (AVn_uh_)	441.1	288.0	−34.7%	368.0	−16.6%	416.7	−5.5%
	Partial hip (AVn_ph_)	299.0	227.7	−23.8%	205.4	−31.3%	217.0	−27.4%
	Hybrid and reverse hybrid (AVn_hrh_)	68.6	37.7	−45.1%	48.0	−30.0%	60.2	−12.2%
	Cemented knee (AVn_ck_)	474.0	267.5	−43.6%	316.5	−33.2%	358.1	−24.4%
Other types of surgery	Spinal segmental implants (AVn_ss_)	1553	1226.4	−21.1%	966.9	−37.7%	970	−37.5%
	Hemicolectomy (AVn_he_)	267.4	175.3	−32.9%	232.6	−22.3%	242.9	−9.1%
	Post-fracture osteosynthesis procedures in the femur (AVn_pf_)	869.1	584.4	−32.7%	774.2	−10.9%	785.2	−9.6%
	Coronary artery bypass grafting (AVn_cb_)	233.1	161.4	−34.4%	216.6	−13.1%	203.3	−12.8%
	Coronary angioplasty (AVn_ca_)	317.3	208.7	−34.2%	270.8	−14.6%	293.7	−7.4%

## Data Availability

The data presented in this study are available on request from the corresponding author.
